# Supplementary material for: Are carbon emissions trading and green financial instruments synergistic? -Comprehensive quantitative research based on content analysis
Source: PLoS One. 2024 Mar 7;19(3):e0298601. doi: 10.1371/journal.pone.0298601 (PMC10919601; doi:10.1371/journal.pone.0298601)
Supplement: S1 Data — (ZIP) [file pone.0298601.s001.zip › Data/地级市Z_F工作报告.zip!/║┌┴·╜¡/╦1⁄2╤╝╔╜╩╨/└┤╫╘╣1⁄2╓┌║┼í╛┬φ┐╦╩2╛▌═°í┐_2015.docx]

各位代表：

现在，我代表市人民政府向大会报告工作，请予审议，并请各位列席的同志提出意见。

一、2014年工作回顾

过去的一年，是我市经济社会发展极为艰难的一年。面对支柱产业市场持续低迷、主导产品量价齐降、融资瓶颈凸显、改善民生和维护稳定压力增大等多重困难交织叠加的严峻形势，我们在市委的领导下，在市人大和市政协的支持下，团结和带领全市人民，沉着应对，攻坚克难，努力克服经济下行带来的不利影响，在困境中保持了民生持续改善、社会安定和谐、各项事业稳步发展的良好局面。

（一）积极应对经济下行，全力维护经济持续发展

一年来，我们加大经济运行监测分析力度，实施多项扶持措施，组织企业千方百计保生产，整体经济下行趋势逐步放缓。预计全市地区生产总值实现460亿元，同比下降11%左右。粮食生产实现“十一连增”。总产达到55.7亿斤，增长7.1%。农村经济总收入实现137亿元，增长5.4%。建设现代农业示范带11条、示范园区45个。新增农业产业化龙头企业9家、标准化规模养殖场8家。新增土地流转面积62万亩、农民专业合作社437个。 农业机械化率提高到92%。建成农村产权交易市场2个。集贤和宝清两县被列为“两大平原”现代农业综合配套改革试点县。骨干工业企业努力保持运行。受经济下行冲击影响，预计规模以上工业增加值实现78亿元，同比下降49%。面对严峻形势，骨干企业顽强拼搏，积极开拓市场、降本增效、筹措资金，保持生产经营稳定。双矿集团在煤炭销量和价格齐降的困境下，产量达到1108万吨，上缴税金4.6亿元。建龙集团完成主营业务收入85.1亿元、利税2.8亿元，实现了扭亏为盈。新时代水泥生产经营逆势上扬，水泥产量111.7万吨、销售收入3.8亿元、利税1.1亿元。服务业稳步发展。预计社会消费品零售总额完成109.7亿元，同比增长9%。旅游总收入实现13.5亿元，同比增长23.9%。饶河县被列为全国旅游标准化试点县。 外贸进出口总值实现13亿美元，同比增长8%。金融行业增加值同比增长13%，金融机构存、贷款余额分别增长10.5%和21.9%。

（二）充分利用政策机遇，推进经济转型发展

传统产业改造步伐加快。煤炭和粮食加工产业向深加工延伸，钢铁产业大力推进循环、清洁生产，建材产业突出高效节能和绿色环保。地方煤矿整治整合全面启动，兼并重组19家主体企业已经确定。市同心橡胶厂等3家企业被认定为省级专利优势创建企业。同鑫面粉、SYS制砖机获驰名商标，两家企业分别获得省级奖励100万元。园区建设得到加强。市经济技术开发区晋升为国家级开发区，启动了总体规划和专项规划编制，基础设施建设加快。四县绿色食品园区和四区中小企业园区开始规划建设。产业项目建设稳步推进。开复工5000万元以上产业项目67个，完成投资63.1亿元;省重点推进的产业项目开复工率、投资完成率均达到100%。建龙LNG等19个项目建成投产；中俄国际文化物流经贸产业园等41个项目开工建设；龙煤天泰10万吨芳烃等26个项目正在加快建设；龙泰60万吨煤制烯烃等6个项目的前期工作取得实质性进展。受整体投资规模缩减的影响，固定资产投资完成150亿元，同比下降66%。向上争取资金力度加大。受主体税源锐减的影响，公共财政预算收入完成18.5亿元，同比下降36.3%；财政支出103.5亿元，同比增长0.6%。为缓解财政支出压力，争取资源枯竭型城市转型、生态治理、老工业区搬迁、城市基础设施建设等政策性资金74.3亿元，有效保障了民生事业发展。

（三）坚持多措并举，持续推进城乡建设

投融资体系不断完善。组建了6个投融资平台，成立了国有资产经营公司，初步建立了借管用还一体化的投融资体系，全年引入社会资本26.8亿元。重点工程建设有序推进。完成市政重点工程21项。安邦河下游治理工程完工，滨水北城核心区基础设施建设基本完工，铁西路、东环路、北环路和卧虹桥、东福桥等拓宽改造工程竣工通车。大和镇至五林洞边防公路、宝密公路友谊段完工，依饶公路双鸭山至集贤段等工程正在加快推进。小城镇建设深入实施。4个省级小城镇建设试点乡镇完成投资3.2亿元，实施建设项目27项。“美丽乡村”建设投入资金8742万元。新建农村样板社区6个，友谊县富强社区被评为“全国和谐社区建设示范单位”。城市管理得到加强。5个弃管小区、197栋弃管单体楼实施了物业管理。改造供热管网49.7公里。开展了春秋两季环境综合整治。颁布实施了清雪令，购置专用清雪设备65台套，市区主要街路机械化清雪基本全覆盖。“天眼”工程二期投入使用。市应急指挥体系高效运转，74家应急联动单位接处警1.5万件。生态保护力度加大。实施工程减排项目45个。市污水处理二期工程主体完工，寒葱沟水库水源污染防治和1.5万亩小流域综合治理工程全面完成。淘汰黄标车和老旧车辆5681台，撤并小锅炉63座。植树造林15.1万亩，森林覆盖率达到40%。饶河县被评为“全国湿地保护工作先进县”。单位GDP综合能耗同比下降2.5%。

（四）把群众利益放在更加突出的位置，努力保障和改善民生

居民住房条件不断改善。煤矿棚改工程竣工住宅5342套，安置回迁居民2798户。城市棚改工程新建住宅1190套，竣工480套。改造农村泥草房1752户、危房2955户。分配廉租住房500套，发放廉租房补贴1830万元。民生保障能力持续增强。全市城镇新增就业2.5万人，城镇登记失业率控制到4.3%以内。转移农村劳动力13.9万人。城乡职工和居民“五项”保险参保人数均高于上年。新农合人均筹资标准由350元提高到390元，重特大疾病报销种类由14种增加到22种。困难群众医疗救助比例提高到65%，最高限额达到2万元。城乡低保标准分别提高10%和18%。全市居家养老服务覆盖面达到75%。多渠道筹措资金近千万元，开展了“1+6”专项济困救助行动。规范了机关事业单位津、补贴。城乡居民收入分别增长5%和10%。社会事业协调发展。市职业教育集团正式成立，黑煤职院、新高级中学投入使用，优化调整偏远薄弱学校116所，新建和改扩建幼儿园42所。 在全省率先建立了“三位一体”医防合作新型结核病防治服务体系。龙江剧《农民的儿子》在龙江东部地区巡演。市县博物馆、群众艺术馆等公共文化设施全部免费开放。举办了“城市之光”等文艺演出40余场。参加省运会获金牌38枚，创青少年记录11项。社会保持和谐稳定。突出抓好安全生产，煤矿、交通、火灾等伤亡人数均控制在省定指标内。深入开展“开门大接访”活动，加大信访矛盾纠纷调处力度，到省、进京非正常访分别下降75%、4.2%。社区矫正覆盖率100%。全市刑事案件发案起数下降4.5%，破案率提高12.5%。统计、质监、工商、档案、史志、地震、气象，以及计生、人防、外事侨务、民族宗教、妇女儿童、残联、红十字等部门礼拜五秘书网，在服务经济社会发展中发挥了积极作用。

（五）切实转变作风，政府自身建设水平得到提高

认真整改“四风”问题。扎实开展党的群众路线教育实践活动，对市政府班子及成员征集的1284条意见和建议，建立了统一台账，逐项明确了整改措施和时限。启动整章建制工作，完善了因公出国、公务接待、差旅费报销和“三公”经费管理等制度。出台了《市政府领导班子成员下基层工作机制》，打通联系服务群众“最后一公里”。民主法治建设得到加强。认真执行市人大及其常委会决议，自觉接受市人大法律监督和市政协民主监督，定期向人大报告、向政协通报重点工作。认真办理人大代表议案、建议和政协委员提案，29件人大代表意见、建议和95件政协委员提案全部办理完毕。规范性文件备案率及审核率均达到100%。政府运行更加规范。认真执行重大问题集体决策、重大事项报告、战线分工负责和综合协调工作制度，修订了市政府工作规则，建立了城市规划委员会、土地管理委员会等集体决策机制。行政审批制度改革成效明显。取消、合并、下放行政许可事项280项，精简比例达61%。审批窗口单位受理办结业务24.9万项，同比增长1.9倍。推进商事登记制度改革，实施了“一表制”申报和“一口制”办理的新模式。廉政建设持续深化。认真落实党风廉政建设责任制，履行“一岗双责”。严格执行中央“八项规定”、省委“九项规定”和市委“三个带头、八项注意”规定。大力开展了“十查十治”专项治理。成立市固定资产投资审计中心，强化了政府投资项目审计。推行了行政事业单位公务卡结算制度，严控一般性支出。全面开展了公务用车、办公用房、楼堂馆所和机构编制清理工作。严肃查办违规违纪案件，立案383件，党纪政纪处分429人。

各位代表，一年来的艰苦努力，使我们深切地体会到：之所以我市经济能在挑战中逆势突破、在困境中奋力前行，得益于历届班子打下的坚实基础；得益于市委的坚强领导和市人大、市政协以及社会各界的鼎力支持；得益于全市上下的拼搏实干。在此，我代表市政府，向全市广大干部群众，向给予政府工作大力支持的人大代表、政协委员，各民主党派、工商联、无党派人士和人民团体，向驻双部队、武警官兵和公安民警，向在双中省直单位和所有关心支持双鸭山发展的各界人士，表示衷心的感谢！

实事求是地讲，虽然我们付出了比以往更多的努力，但总体发展的成果仍不理想。部分经济指标增速大幅回落，既有受经济下行大环境的影响，也有理性回归的因素；一些重点市政和民生工程没有完成，既有客观环境对投资的制约，也有我们政府自身努力不到位的问题。我们已经清醒地认识到，当前我市经济社会发展仍然存在着许多矛盾和问题：一是结构性矛盾愈加突出。传统产业比重仍然偏大，粮食加工和煤炭两个产业占工业的比重超过60%，特别是“原字号”产品占比过高的问题尤为突出，导致我市主导产品缺乏市场竞争力。二是财力保障难度增大。2014年市本级公共财政预算收入不足10亿元，已经回落到2008年水平，而刚性支出却达到了25亿元，是2008年的2倍多。加快城市建设和民生改善所形成的政府性债务，也进入了偿还高峰期，财政支出压力增大。三是民生改善的任务依然繁重。沉陷区治理和各类棚户区改造压力巨大，市区交通堵塞、燃气和供水不足、城区排涝不畅、环境脏乱差、小区弃管和房照办理等问题亟待解决。四是发展后劲不足。困境下企业投资力度减弱，产业项目储备不多，在建项目投资规模不大，战略性新兴产业项目缺乏。部分企业工艺技术改造和产品研发创新意识不强，对未来的发展形成了一定制约。五是安全生产的基础性工作不牢固。政府关于安全工作的要求和措施在个别部门和企业没有落实落靠，仍存在诸多安全隐患，个别煤矿安全矿长挂名、驻矿监督员工作顺拐，特别是新久煤矿瓦斯事故的发生，给人民群众的生命财产造成了很大损失。六是政府还不能完全适应转型发展需要。部分政府工作人员的改革勇气不足、市场意识不强、法治精神不够、群众观念不牢，“四风”问题在个别政府部门和领导干部中依然存在。这些问题，我们将高度重视并认真加以解决。

二、2015年工作安排

2015年是全面深化改革的关键之年、依法治国的启动之年、“十二五”规划的收官之年，也是我市推进城市转型发展的攻坚之年。做好今年的工作，要求我们必须正确判断形势，准确把握新常态。新常态是中央对我国经济发展阶段性特征做出的一个战略判断。新常态下，经济增长速度、发展方式、发展动力、经济结构将发生深度调整和变化，我市经济发展阶段性特征和趋势也将随之改变。从增长速度看，受宏观环境影响，将从高速向中低速或低速转变。从发展方式看，将从“原字号”、“规模扩张”粗放式发展，向以质量效益为核心，盘活存量、做优增量、产业升级的集约式发展转变。从发展动力看，将从单纯依靠要素驱动，向以要素驱动为基础，依靠深化改革和技术创新转变。从经济结构看，将从传统资源能源为主，向资源能源产业升级、服务业加快扩张转变。粮食向绿色食品产业延伸；煤炭向高端产业转化；钢铁向综合循环扩能升级；服务业向以对俄贸易为重点，生活性、生产性服务业全面扩张。

适应新常态，加快转型发展，我们既面临着难得的机遇，又面临着严峻的挑战。从机遇看，国家实施“一带一路”战略、建设“中蒙俄经济走廊”、继续支持东北老工业基地振兴和资源型城市转型，以及我省深入实施“五大规划”发展战略、建设“龙江陆海丝绸之路经济带”、稳增长65条、煤炭城市转型发展规划，为我们转变发展方式、加快转型发展，提供了强有力的支撑和不可多得的机遇。从挑战看，世界经济复苏缓慢，国内和我省经济下行趋势不会明显改变，经济转型发展已成大势。特别是与我市息息相关的煤炭、钢铁产业，市场低迷、刚性需求不足的状况短期内难以扭转。单纯的等靠、维持必将在产业转型升级过程中被淘汰，转型发展是必由之路。差距就是潜力，困难就是动力。越是艰难时期，越是紧要关头，我们越要保持定力、提振信心，越要抢抓机遇，奋发有为。

今年工作的总体要求是：深入贯彻落实党的十八届三中四中全会、省委经济工作会议和市委十届五次全会精神，主动适应经济发展新常态，坚持市委确定的“基础在煤炭、潜力在粮食、发展在电化、提升在贸易、增长在钢铁”经济发展思路，牢牢把握稳中求进的总基调，以提高质量和效益为核心，以推进转型发展为主线，以深化改革和科技创新为驱动，以依法治市为保障，全力转方式、调结构、稳增长，强化风险防控，努力改善民生，多做打基础、利长远的实事，推动经济平稳健康发展和社会和谐稳定。

主要预期目标：地区生产总值增长3%左右，规模以上工业增加值增长2.5%，公共财政预算收入增长2%，固定资产投资增长6%以上，社会消费品零售总额增长8%，外贸进出口总值增长2%，城乡居民收入分别增长5%、7%以上，单位GDP综合能耗下降3.7%。

（一）坚持在保运行中推动产业升级，为转型发展奠定基础

把保持经济稳定运行作为首要任务，以质量和效益为核心，加快做优一产、提升二产、壮大三产的产业升级步伐。

大力发展优质高效农业。加快调整种植结构。“调玉米、稳水稻、强大豆”，推广主栽品种，推进标准化生产、合作化发展。粮食总产稳定在55亿斤以上；新建500亩以上家庭农场5个、万亩农民合作社15个；农业机械化率达到93%。加快推进畜牧业规模化、产业化发展。新建高标准肉牛、生猪规模养殖场10个和15个；新上畜牧产业化龙头企业4户；畜牧业产值增长10%。加快农业基础设施建设。抓好大佳河灌区配套改造和靠山高标准基本农田整治；积极做好“三江连通”工程项目的前期服务；继续推进大叶沟水库、徕凤湖水库等水源项目前期工作；全力做好6.8万亩节水增粮行动工程建设。抓好合作社与企业联结，推进产加销一体化。重点扶持恒大等绿色龙头企业发展，叫响绿色、有机品牌，加快建立农产品安全保障体系，把绿色、有机食品产业做大做强。

巩固提升工业经济。帮助双矿集团、建龙钢铁等重点企业搞好产销对接，指导企业创新营销模式，巩固域内市场份额，拓展域外和境外市场，扩大市场保经营。推进地方煤矿整治整合和各类停产半停产企业开工，启动存量保运行。以市场需求为导向，引导企业加快产品创新、工艺提升，延伸产业链条，实施信息化改造，降本增效稳增长。强化生产要素协调，在推进银企对接的同时，引导企业树立资本运营意识，通过资产证券化等方式，扩大直接融资比例，为企业发展提供资金支撑。保障重点企业生产原料和产品运输，帮助发电企业争取上网电量，积极争取直供电政策支持。依托市开发区和各县区园区，高起点打造中小微企业“加速器”。建立中小微企业公共服务平台，完善激励政策，全年新增中小微企业100户左右。“盘活闲量，升级存量，引进增量”，逐步建立起以煤电化、冶金循环、绿色食品加工、新能源和新材料为重点的现代工业体系，逐步提高非煤产业占工业经济的比重。

加快发展服务业。把对俄贸易战略升级放在首位，加快中俄国际文化物流经贸产业园大平台、“双鸭山中俄国际城”等载体建设，全年对俄进出口总值增长20%以上。以改造提升传统商贸服务业为基础，加快综合服务体建设，确保欧蓓莎小商品交易中心、联丰中俄商贸中心、宝清新农大市场建成运营。完善布局，优化环境，加快发展现代物流。大力推广互联化服务方式，发展电子商务，带动商贸服务业发展方式的快速提升。深度挖掘森林、湿地、农业观光、城郊休闲、历史文化等旅游资源潜力，加快重点景区和城市国际旅游配套设施建设。支持组建跨区域旅行社团或联盟，打造6条跨区域和5条域内精品旅游线路，推动旅游产业升级。高起点制定养老服务产业行业标准，重点发展规模较大、功能配套、“医养”结合、绿色生态的养老服务项目。积极拓展“候鸟式”养老，发展养老地产。把金融业作为重点产业培育，大力引进股份制银行、非银行类金融机构，加快组建地方金融机构，促进保险、证券业健康发展，发展普惠金融，优化金融生态环境，防范金融风险。适应消费需求，加快发展通信、文化、体育、健康等产业。

（二）狠抓质量效益型产业项目建设，加快转型发展步伐

全力抓好以“准备之冬”、“开工之春”、“建设之夏”、“竣工之秋”为重点的产业项目提升年活动。

加大招商引资力度。面向发达地区、产业转移重点地区，以及与我市产业发展方向高度契合的区域，紧盯央企、大型民营尤其是500强企业，采取资源和特许经营权招标招商、科技成果和专利产业化招商、小分队定向招商、发达地区商会招商、以商招商和友情招商等办法，大力引进投资规模大、科技含量高、市场前景好、财税贡献多的质量效益型产业项目。全年引进5000万元以上产业项目100个，招商引资到位资金200亿元。

推进产业项目建设。统筹传统和新兴产业发展，重点推进煤电化、对俄贸易、绿色食品、冶金循环产业项目开发建设。推进建龙160万吨干熄焦和6.5万千瓦高温超高压发电、新时代水泥80万吨工业废渣和余热综合利用、双矿东荣三矿扩建、恒大有机大豆油扩能等项目尽快开工，神华国能2×60万千瓦电厂、集贤吴越科技园玉米肽、三聚华本煤化工、广汇三江汽车城等项目加快建设，龙煤天泰10万吨煤制芳烃、中俄国际文化物流经贸产业园、友谊宝盟粮食深加工等项目竣工投产；加快建龙300万吨钢铁扩能、鸿森1000万吨褐煤热解提质、双锅移动式秸秆成粒生产线等项目前期；确保龙泰60万吨煤制烯烃、双矿2×35万千瓦低热值煤电厂等项目完成核准。全市必保开复工5000万元以上产业项目100个，谋划储备100个；竣工投产项目占项目总数30%以上；产业项目投资占固定资产投资比重65%以上。

加快园区建设步伐。理顺市开发区工作体制机制，完善招商、投资和综合服务职能，完成总体规划和专项规划编制。重点推进中俄国际文化物流经贸产业园和煤化工产业园配套基础设施建设，确保企业孵化中心、消防站、变电站投入使用。加快四个县绿色食品产业园和四个区中小企业园的建设。大力推进项目向园区集中，产业向园区集聚，必保市开发区开复工5000万元以上产业项目10个，各县绿色食品产业园开复工项目5个，各区中小企业园开复工项目3个。

深入推进区域合作。结合双矿集团深化改革，推进四个区和集贤县与双矿集团在地方煤矿托管、机电产品开发、畜牧业和养老产业发展等方面紧密合作，加快盘活闲量、做优存量，分流富余人员。着眼森工转型发展，推进集贤、宝清、饶河三个县和岭东、宝山两个区与森工在食品开发、环艺、旅游、林下经济等方面务实合作。借助农垦高效农业和绿色食品带动优势，推进四个县和宝山区与农垦在农业规模化生产、集约化经营、绿色食品开发等方面深化合作，共同培育优势主导产业，推进区域融合发展、一体发展。

科学谋划、组织编制我市“十三五”规划，指导我市未来五年经济社会发展。

（三）加快城乡基础设施建设，为转型发展创造条件

抢抓国家扶持基础设施建设的重大机遇，扎实推进“山水生态城”建设。

推进基础设施建设。确保市滨水北城人工湖公园、体育公园投入使用，康泰路、平安街全部完工，民生北路和二马路延伸段开工建设。启动东环路南段、建设路、新城路、迎宾大道、九阳路等道路的改造工程。如期推进依饶公路双鸭山至集贤段和太保段、饶河北出口公路、宝清绕城公路、危桥改造、农村路网改善和市县客运中心工程等项目建设。积极推进双佳铁路扩能改造、佳木斯机场迁建、饶河界江大桥和双宝高速公路等项目前期工作。

着力建设宜居环境。积极争取沉陷区改造政策，推进保障性安居工程建设。煤矿棚改竣工住宅6000套，城市棚改开竣工住宅500套，力争南市区棚改工程完工。继续推进时代新城建设。启动实施弃管小区环境、城区交通环境、农村人居环境的专项整治和老城区绿化、农村饮水安全等专项工程。

完善城市管理服务。编制和实施城市交通、防涝排涝等专项规划。推进公共设施管护、消防设施配套，以及环卫保洁、市容监管和物业管理。继续实施老旧供热管网和寒葱沟供水项目改造。完善老系统，开辟新的燃气气源。规范引导房地产和商业综合体有序发展。加强城市综合执法，完善配套服务功能，推进网格化、数字化和精细化城市管理礼拜五秘书网。

加大生态建设力度。继续推进水源地、林地、湿地和矿产开发区域生态恢复与保护。加强安邦河流域水环境综合整治和市区周边山体绿化。落实“三条红线”水资源管理制度，编制水资源综合利用规划。推进植树造林，新增5.25万亩。推进节能减排，强化污染防治，严格执行新上项目“三同时”制度。抓好秸秆禁烧、淘汰黄标车和老旧车辆及燃煤小锅炉治理工作。完成污水处理二期工程和污水管网改造，加快建龙等重点企业减排措施的实施。（四）加快发展民生事业，让转型发展的成果惠及百姓

按照保基本、兜底线、促公平、可持续的原则，发展民生事业。

抓好就业创业工作。统筹城乡就业，完善扶持政策、保障机制和服务体系，抓好高校毕业生、退伍军人、企业下岗困难职工、城镇弱势群体就业。突出抓好援企稳岗工作，稳定就业形势。加快创业基地建设，扩大就业渠道。力争城镇新增就业2万人，城镇登记失业率控制在4.3%以内。

强化社会保障。继续扩大城镇职工和居民“五项”保险覆盖面。推进基本医疗保险市级统筹，城镇职工和居民医疗保险待遇封顶线分别提高1万元，失业保险待遇提高到700元，继续提高生育保险待遇。新农合人均筹资标准由390元提高到450元，报销待遇比例提高5%。完善养老服务体系，确保每千名老年人拥有养老床位30张。加快居家养老服务体系建设。提高城乡低保和农村五保供养标准，继续开展“1+6”专项济困救助行动，深入开展扶老、救孤、助残等救助工作，大力支持慈善、捐赠、福彩和群众互助等社会公益活动。加大劳动监察力度，切实维护劳动者特别是农民工的合法权益。

优先发展教育事业。科学编制学区规划，统筹调整教育网点。启动义务教育薄弱学校改造工程，九年一贯制实验学校、十八中新建等项目投入使用，五中改扩建项目主体竣工，铁西小学项目完成前期工作。加大公办幼儿园建设力度，滨水北城公立幼儿园投入使用，提高市辖区公办示范幼儿园办学水平。鼓励引导社会力量办学，规范发展学前教育。继续推进义务教育标准化学校建设，达标率达到80%以上。严格规范办学行为，强化师德、师风教育。改善黑煤职院、职教集团办学条件，提高办学水平，为我市经济社会发展提供人才支持。

稳步发展卫生事业。确保新人民医院投入使用，精神病院完成主体工程，启动中医院和妇幼保健院改扩建工程。继续深化医疗卫生体制改革，完善基本药物制度，加快建立分级诊疗制度。强化疾病防控、卫生综合监督和应急管理能力。充分发挥质控中心作用，提高医技水平和医疗服务质量。建立医调委第三方调解机制，实施医疗机构不良积分管理办法，净化医疗市场。

大力发展文体事业。确保市文广中心投入使用。落实文化惠民工程，以“百馆千站”送文化下基层活动为载体，推进“基层文化服务年”活动，抓好文化信息资源共享基层网点和图书流动站建设。加大文化遗址保护力度，积极申报非物质文化遗产项目。广泛开展全民健身运动，加快竞技体育发展，满足人民群众多样化的体育需求。

继续做好广播电视、统计、审计、国土、工商、质监、地方志、档案等工作，关心支持老龄、残疾人和妇女儿童事业，促进民族宗教、外事侨务、地震、气象、人防等各项事业协调发展。

（五）扎实推进改革创新，为转型发展增添动力

深化重点领域和关键环节改革，着力推进技术创新，不断增强经济发展的活力和动力。

深化行政体制改革。扎实推进机构改革，优化政府机构设置、职能配置，严控机构编制。继续推进事业单位分类改革，探索医疗、科技等机构的用人机制改革。深化行政审批制度改革，推行“一口受理”，实行“限时办理”，严格“规范办理”，坚持“透明办理”，推进“网上办理”，建立政府权力清单、责任清单制度，力争审批事项再减少20%以上。深化综合执法体制改革，推进重点领域联合执法。深化收费体制改革，取消、合并一批行政事业收费，推行“一口制、一次性”收费。以特许经营权、矿产资源为重点，加快市场化配置资源机制改革。适时启动公务用车改革。

深化财政体制改革。明确公共预算、政府性基金预算、国有资本经营预算、社会保险基金预算收支范围，适时建立三年滚动中期财政预算体制。将政府债务纳入预算，严格规范债务借、用管理，防范债务风险。清理税费优惠，规范财政税收优惠政策。全面强化税收征管和非税征缴。完善支出标准，改革支出方式，建立健全政府投资管理体系。

深化国有企业改革。理顺国有企业和经营性事业单位管理体制，建立以资产为核心的管理体系。推进产权制度改革，建立自主经营、权责清晰的经营核算机制和法人治理体系。积极稳妥推进国有大集体改革。

深化农业综合配套改革。搞好集贤保胜村、宝清东强村和永乐村土地承包经营权确权登记试点，推进农村产权交易中心建设，土地流转面积达到230万亩。加快各类涉农金融服务体系建设，抓好宝清土地承包经营权抵押贷款试点。建立农业保险竞争机制，提升保障水平，农业保险参保面积达到180万亩。

加快科技创新。以技术创新为核心，积极培育以企业为主体、科研院所为支撑的技术研发平台和创新联盟。加强科技成果的引入、推介、招商和转化对接，依托市县开发区，高起点打造科技创新孵化器和创业孵化基地，推进技术成果产业化。鼓励“小改小革”，引导和扶持产业工人进行技术创新。加强专业技术人才的引进和培养，力争年内完成黑煤职院煤化工研发基地和以建龙等企业为重点的博士后工作站建设。

（六）全力抓好社会稳定，为转型发展提供保障

完善社会管理机制，提升管理水平，营造安定和谐的社会环境。

强化社会管理。抓好村委会的换届选举工作，争创全国农村社区建设全覆盖先进县区。实施社区基础建设“双百示范工程”，建设城市示范社区7个、农村示范社区6个。开展“百项服务进社区”活动，推动公共服务向社区村屯延伸。积极稳妥地推进户籍改革。深入推进“平安双鸭山”建设，完善社会治安防控体系，推进公安信息化建设，加大治安突出问题整治力度，提高反恐和应急处置能力，依法严厉打击各类违法犯罪。加大普法力度，深入开展“法律六进”活动。

全力确保安全生产。认真履行安全生产“一岗双责、党政同责、齐抓共管”和“三个必须”要求，强化主体责任、监管责任和领导责任，夯实安全生产基础。以煤矿安全生产为重点，加快推进地方煤矿整治整合。全面加强非煤矿山、道路交通、消防、食品药品、危险化学品、烟花爆竹、森林防火、防汛抗旱和特种设备等行业安全监管，强化源头管理，开展专项整治，最大限度地减少一般事故、控制较大事故、防范和遏制重特大事故发生。

做好信访稳定工作。认真落实信访工作责任制、信访接待日和重点案件领导包案制度。完善人民调解、司法调解和行政调解联动机制，建立畅通、规范、有序的群众诉求表达、利益协调和权益保障渠道，引导群众依法有序信访，妥善化解社会矛盾。

（七）加强政府自身建设，提高服务转型发展的能力

按照“为民务实清廉”的总要求，努力建设法治政府和服务政府。

坚持依法行政。以法治思维和方式履行政府职能，解决处理矛盾和问题，做到法定职责必须为、法无授权不可为。自觉接受人大及其常委会的法律监督、工作监督和政协的民主监督,切实做好议案、建议、意见和提案的办理工作，虚心听取各民主党派、工商联、无党派人士和社会各界的意见。完善政府法律顾问制度，规范决策权限和程序，建立重大决策公众参与、专家论证、风险评估、合法性审查和集体讨论决定的决策机制。加强执法监督，完善执法程序，规范行政许可、行政处罚等执法行为。加强行政执法队伍建设，提升行政执法队伍的法治意识、职业素养和专业水平，做到严格规范公正文明执法。

改进工作作风。加强政府信息平台和公开机制建设，落实政府及部门新闻发言人制度，重大决策部署和重要事项必须及时向社会公开，保障群众的知情权、监督权。密切联系群众，畅通市长热线、市长信箱，落实领导干部下基层工作机制，有效解决服务群众“最后一公里”问题。大力倡导务实作风，严控发文和会议数量，坚持开短会、讲短话，严控各类检查、培训、评比和表彰活动，把更多时间花在倾听民意、体察民情、为民解忧上，把更大功夫下在谋划工作、解决问题、狠抓落实上。深入开展“十查十治”专项治理，持续整治“四风”，健全改进作风常态化制度。加强政府绩效管理，强化政府部门履职评估、行政问责和效能监察，提升行政效率和质量。

加强廉政建设。认真落实党风廉政建设责任制，切实履行“一岗双责”，各级政府领导干部带头遵守中央“八项规定”、省委“九项规定”、市委“三个带头、八项注意”和廉洁自律各项规定。认真执行厉行节约反对浪费的规定，严控一般性支出，确保“三公”经费只减不增。加强对重要部门、关键岗位人、财、物的监督管理，强化资金使用、工程建设、政府采购等重点领域的行政监察和审计监督，规范权力运行。加强反腐倡廉制度创新，积极推进廉政文化建设，健全惩治和预防腐败体系，强化廉政风险防控。加大纠风和专项治理力度，坚决查处权力寻租、权钱交易和损害群众利益的行为。

各位代表，面对困难和挑战，我们必须勇于担当，迎难而上。让我们在市委的坚强领导下，在市人大、政协的监督支持下，提振精神，凝聚力量，开拓创新，奋勇拼搏，扎实做好今年经济社会发展各项工作，努力谱写双鸭山人民美好生活新篇章！
